# Do the Changes in Hip Lateralization Up to One-Year Post-reduction in Developmental Dysplasia of the Hip Affect Those Observed at the Age of Four?

**DOI:** 10.7759/cureus.99371

**Published:** 2025-12-16

**Authors:** Yohei Tomaru, Takashi Saisu, Makoto Kamegaya, Yasuhiro Oikawa, Akitoshi Sakuma, Jun Kakizaki, Yuko Segawa, Yuta Tsukagoshi, Daisuke Nozawa, Hiroshi Kamada

**Affiliations:** 1 Department of Orthopaedic Surgery, Chiba Child and Adult Orthopaedic Clinic, Chiba, JPN; 2 Department of Orthopaedic Surgery, Chiba Children’s Hospital, Chiba, JPN; 3 Department of Pediatric Orthopaedic Surgery, University of Tsukuba, Tsukuba, JPN; 4 Department of Orthopaedic Surgery, Ibaraki Children’s Hospital, Mito, JPN; 5 Department of Orthopaedic Surgery, University of Tsukuba, Tsukuba, JPN

**Keywords:** center head distance discrepancy, closed reduction of hip, developmental dysplasia of the hip (ddh), lateralization, pavlik harness

## Abstract

Background

Treatment outcomes of developmental dysplasia of the hip (DDH) are evaluated by various factors. Although it is reported that centripetality is important for the development of the acetabulum in DDH, only a limited number of studies have examined the changes in lateralization following reduction. This study aimed to determine whether variations in hip lateralization within one year after reduction with the Pavlik harness (PH) in DDH influence the degree of lateralization observed at a mean age of four.

Methodology

This study included DDH patients who were successfully treated exclusively with the PH between 2014 and 2020, and who were able to be followed up for more than three years. For the evaluation of lateralization, the center head distance discrepancy (CHDD) was utilized. Measurements of CHDD were collected at four key time points: before the application of the PH (CHDD-before), immediately following its removal (CHDD-just after), one year post-reduction (CHDD-1y), and at a mean age of four (CHDD-4y).

Results

In total, 23 patients (one boy and 22 girls) were included in this study. The mean ages at the time of each CHDD measurement were 4.2 months (range = 1.4-6.2 months), 7.2 months (range = 4.4-9.2), 1.7 years (range = 1.3-2.4), and 4.9 years (range = 3.1-8.2). The average CHDD values were 18.5% (CHDD-before), 5.3% (CHDD-just after), 3.2% (CHDD-1y), and 2.5% (CHDD-4y). Only ΔCHDD (from one year to four years) showed a significant correlation with CHDD-4y (Pearson’s correlation coefficient, r = 0.56, p < 0.01).

Conclusions

Some cases that showed improvement within the first year after reduction later deteriorated, while others that initially worsened within the first year subsequently improved. Changes in CHDD that occurred more than one year after reduction were found to influence the outcomes at a mean age of four years. Therefore, we suggest a follow-up period of at least four years is necessary for an adequate assessment.

## Introduction

The treatment outcomes for developmental dysplasia of the hip (DDH) are evaluated and affected by various factors, including the severity of dislocation, acetabular index (AI), center head distance discrepancy (CHDD), the age at reduction, and the method used for reduction, among others [1-5]. It is reported that centripetality is important for the development of the acetabulum in DDH cases [5-8]. A lack of proper hip joint stability, which alters stress distribution between the acetabulum and the femoral head, significantly raises the risk of early osteoarthritis [9-11]. Lateralization of the femoral head following reduction with the Pavlik harness (PH) can occur in cases of DDH. This lateralization is relatively common, with reported occurrences ranging from 7% to 50% of DDH cases post-PH application [12-14].

While the majority of lateralization cases resolve on their own, some do not improve without intervention [12,13,15]. It is not clearly established how much spontaneous improvement can be expected in terms of age and degree of lateralization, nor at what point therapeutic intervention becomes necessary. Furthermore, there is often ambiguity regarding the appropriate treatment for residual lateralization following PH use.

To our knowledge, only a limited number of studies have examined the changes in lateralization following reduction. These reports indicate that, while spontaneous improvement can occur, the outcomes vary considerably among patients. Furthermore, there is a lack of clear guidelines regarding the thresholds for intervention, highlighting the need for more comprehensive research in this area [15,16]. This study seeks to address existing knowledge gaps by analyzing the changes in hip lateralization over time, providing insights that may help develop more standardized treatment protocols for patients with DDH who experience lateralization following reduction by PH. The ultimate goal is to improve long-term outcomes and decrease the incidence of complications, such as early-onset osteoarthritis, in affected individuals. As an initial step, this study focuses on the natural history of lateralization after PH.

This study is designed to enhance the understanding of the potential for spontaneous improvement versus the necessity for active treatment in managing lateralization. By clarifying these aspects, it will support clinicians in making informed decisions regarding the management of residual lateralization following treatment with the PH.

This study aimed to determine whether variations in hip lateralization, represented by CHDD, within one year after reduction with the PH in DDH, influence the degree of lateralization with/without AI observed at a mean age of four years, when additional surgical treatments might be considered to prevent further osteoarthritis.

## Materials and methods

This study included patients diagnosed with DDH who were successfully treated exclusively with PH between 2014 and 2020, and who were able to be followed up for more than three years. Cases of DDH defined as Graf classification greater than class 3, International Hip Dysplasia Institute classification greater than class 3, and/or hip with a positive Barlow/Ortolani sign were included [17,18]. Cases that did not respond to treatment with PH, as well as those receiving supplementary treatments, such as the use of abductor braces and involving teratologic or paralytic dislocations, were excluded from the study. For the evaluation of lateralization and acetabular development, the CHDD and AI were utilized [5,19]. Measurements of CHDD were collected at the following four key time points: before the application of the PH (CHDD-before), immediately following its removal (CHDD-just after), one year post-reduction (CHDD-1y), and at a mean age of four (CHDD-4y). AI was measured at a mean age of four (AI-4y). Two authors independently conducted these measurements twice for each time point to ensure accuracy. The patients were then categorized into the following two groups based on CHDD change: the Deteriorating Group (Group D), characterized by worsening CHDD from just after the removal of the PH to one year later, and the Improving Group (Group I), where CHDD improved over the same timeframe. The CHDD-4y was compared between the two groups using the Mann-Whitney U test.

Additionally, Pearson’s correlation coefficients were calculated among CHDD-before, CHDD-just after, CHDD-1y, CHDD-4y, as well as ΔCHDD-earlier (just after to one year) and ΔCHDD-later (one year to four years). Further, Pearson’s correlation coefficients were calculated between AI-4y and each CHDD Group.

To evaluate the reliability of the CHDD and AI measurements, both the intraclass correlation coefficient (ICC (1,2)) and the interclass correlation coefficient (ICC (2,1)) were calculated. All statistical analyses were conducted using R Studio (R Foundation for Statistical Computing, Vienna, Austria; Version 1.2.5042). A p-value of less than 0.05 was considered statistically significant.

## Results

The study included a total of 27 patients. Among these, three patients were excluded due to insufficient follow-up periods, and one case was excluded because the child underwent additional treatment with an abductor brace. Ultimately, 23 patients (one boy and 22 girls) were included in the final analysis.

The mean ages at the first visit, PH removal, CHDD-1y, and CHDD-4y were 4.2 months (range = 1.4-6.2 months), 7.2 months (range = 4.4-9.2), 1.7 years (range = 1.3-2.4), and 4.9 years (range = 3.1-8.2).

**Figure 1:**
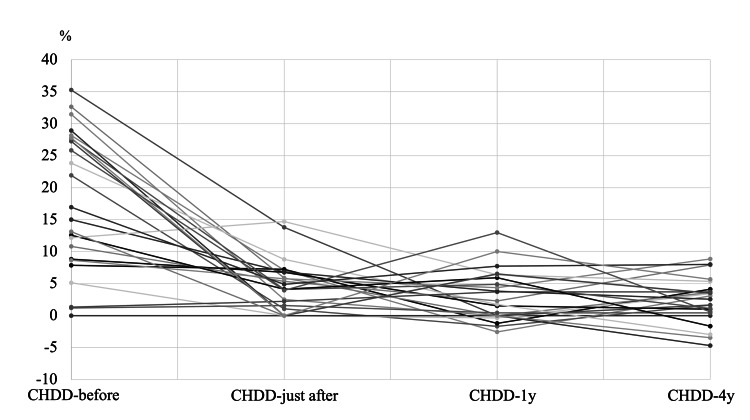
CHDD at four time points in all patients. CHDD at four time points in all patients (before applying PH: CHDD-before, just after removing Pavlik Harness: CHDD-just after, one year after the removal of Pavlik Harness: CHDD-1y, CHDD at a mean age of four: CHDD-4y). CHDD = center head distance discrepancy; PH = Pavlik harness

**Figure 2:**
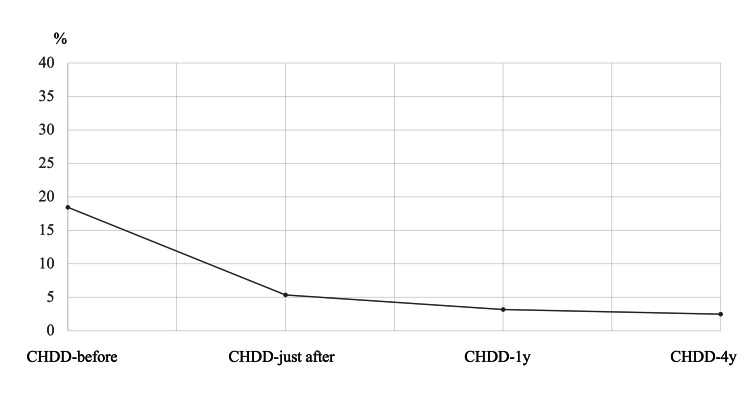
Mean CHDD over four time points in all patients. CHDD = center head distance discrepancy

**Figure 3:**
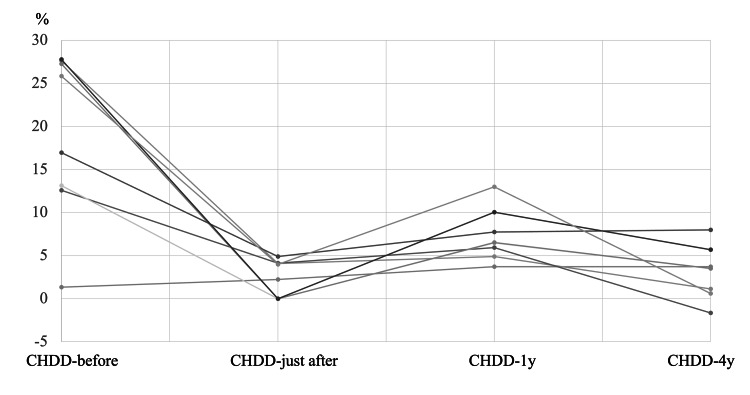
Mean CHDD over four time points in all seven patients in Group D. CHDD is deteriorating between CHDD-just after and CHDD-1y. CHDD = center head distance discrepancy

**Figure 4:**
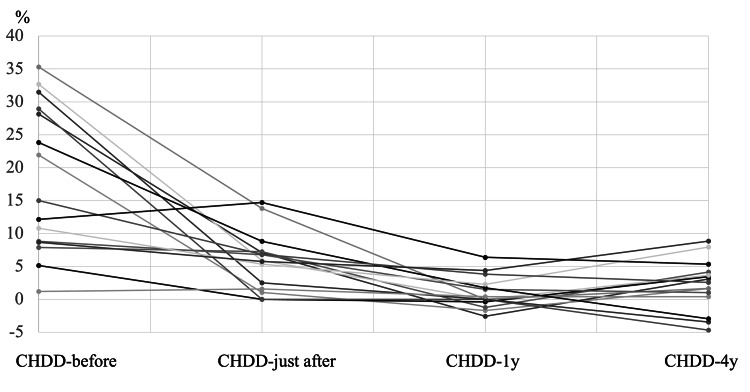
Mean CHDD over four time points in all 16 patients in Group I. CHDD is improving between CHDD-just after and CHDD-1y. CHDD = center head distance discrepancy

The average CHDD values were 18.5% (CHDD-before), 5.3% (CHDD-just after), 3.2% (CHDD-1y), and 2.5% (CHDD-4y) (Figures 1, 2). In Group D, a total of seven hips were included, while Group I included 16 hips. The changes in CHDD for each group are illustrated in Figure 3 and Figure 4.

The CHDD at four years (CHDD-4y) was 3.3% for Group D and 2.3% for Group I. However, there was no significant difference in CHDD-4y between the two groups (p = 0.34, Mann-Whitney U test). Pearson’s correlation coefficients among the CHDD measurements are presented in Table 1. CHDD-4y exhibited a correlation solely with ΔCHDD-later (p < 0.01), whereas no significant correlation was observed with ΔCHDD-earlier (p > 0.01).

**Table 1:** Pearson’s correlation coefficient (r) among CHDD. Pearson’s correlation coefficient (r) among CHDD. Only CHDD-4y and ΔCHDD (1y-4y) significantly correlated. CHDD = center head distance discrepancy

CHDD	before PH	just after PH	1y	ΔCHDD (just after → 4y)	ΔCHDD (1y → 4y)
just after PH	0.28				
1y	0.25	0.33			
ΔCHDD (just after → 4y)	-0.05	-0.63	0.10		
ΔCHDD (1y → 4y)	-0.39	-0.12	-0.77	-0.53	
4y	-0.29	0.24	0.10	-0.13	0.56*

In the most severe cases of CHDD at one year of age, the rate was 13%, but this improved to 0.6% by the age of four. Conversely, two patients with a CHDD of 6% or greater at four years of age had CHDD values of 2.9% and 4.3% at one year of age, respectively.

AI-4y (AI at a mean age of four) was 23.9°(range = 12°-34°). AI-4y and CHDDs did not significantly correlate. ICC (1,2) and ICC (2,1) of CHDD were 0.78 (0.56-0.86) and 0.68 (0.37- 0.85), respectively. ICC (1,2) and ICC (2,1) of AI were 0.95 (0.93-0.98) and 0.89 (0.75-0.95).

Case 1

**Figure 5:**
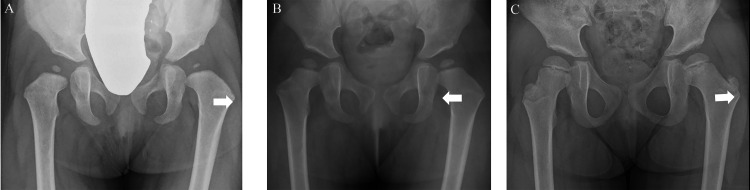
Eight-month-old female with left-sided DDH in Group I. CHDD-just after (A), CHDD-1y (B), and CHDD-4y (C) were 7.5%, -1.5%, and 7.9%, respectively. CHDD = center head distance discrepancy; DDH = developmental dysplasia of the hip

This case involves a left-sided DDH in Group I. The CHDD improved immediately after the removal of the PH and continued to improve until the patient reached one year of age; however, it worsened by the age of four (Figure 5).

Case 2

**Figure 6:**
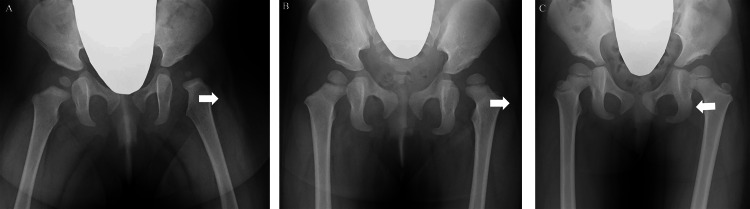
Eight-month-old female with left-sided DDH in Group D. CHDD-just after (A), CHDD-1y (B), and CHDD-4y (C) were 2.4%, 10.0%, and 1.6%, respectively. CHDD = center head distance discrepancy; DDH = developmental dysplasia of the hip

This case presents a left-sided DDH in Group D. The CHDD deteriorated immediately following the removal of the PH and continued to decline until the patient reached one year of age; however, an improvement was noted by the age of four (Figure 6).

## Discussion

In the present study, we observed that the measurement of CHDD at four years of age (CHDD-4y) showed a significant correlation solely with the change in CHDD from one year to four years of age (ΔCHDD (1y-4y)). In contrast, CHDD-4y did not show a significant correlation with the CHDD measurement taken at one year of age (CHDD-1y).

This suggests that lateralization up to one year post-reduction is an unpredictable factor for the CHDD-4y. The improvement in CHDD between the ages of one and four years is a crucial factor that requires careful consideration and further investigation. However, the changes in CHDD between the ages of one and four are important for predicting CHDD-4y. The results indicate that the need for follow-up after one year of reduction is necessary. A variety of factors contributing to lateralization have been extensively reported in the literature. These factors include, but are not limited to, a thickened labrum, increased thickness of the transverse ligament, weakness in the hip abductor muscles, presence of pulvinar, excessive thickness of the cartilage lining the acetabular floor, conditions such as coxa magna, and varying degrees of acetabular dysplasia. Each of these elements can play a significant role in the overall development and stability of the hip joint, influencing the degree of lateralization observed in patients [20-23].

One potential explanation for the lack of a significant correlation between lateralization at one year of age and that at four years may be the dynamic nature of hip stability post-reduction. Even when further development is noted after the reduction procedure, the hip joint can remain unstable for a period of time, particularly around one year post-reduction. During this phase, mild lateralization may still be observed. As children begin to walk, typically after one year of age, the increased muscle power around the hip with weight-bearing force, especially the hip abductor muscle, could contribute to compressing the labrum, cartilage, and pulvinar, thereby promoting improvement in centripetal orientation of the hip joint. It results in further support of the hip stability and the improvement of lateralization.

Based on the findings from this study, alongside our clinical experience with a particular case where the CHDD was recorded at 13% at one year of age and showed improvement by four years, we propose that careful observation is key in managing lateralization concerns for cases with CHDD up to 13% observed one year following reduction.

On the other hand, it is important to emphasize that among those with a normal CHDD-1y measurement (defined as CHDD < 6%), two hips out of a total of 21 (representing 9.5%) were found to have progressed to a CHDD-4y of 6% or more at the age of four. This finding underscores the necessity for careful monitoring, as even normal initial measurements may not guarantee long-term stability and normal hip development.

Furthermore, studies have indicated that lateralization associated with residual labral retroversion in DDH cases typically demonstrates less improvement over time [24]. Therefore, in patients over the age of four exhibiting persistent lateralization, a thorough evaluation becomes crucial. This evaluation should include imaging modalities such as MRI or arthrography to better assess the underlying structural changes [24]. Should lateralization persist beyond the age of four, surgical interventions, including open reductions and/or innominate osteotomy, may be necessary depending on the severity and underlying causes of the observed lateralization. Kim et al. have recommended surgical intervention when the CHDD reaches levels greater than or equal to 6% and when the sourcil shows an upward trajectory in children aged four to five [25]. This condition might coincide with our Case 1 in which 7.9% of CHDD was observed in the CHDD-4y with the upward sourcil. Fu et al. also identified that a Reimer’s index surpassing 38% at age four serves as a significant indication for surgical correction [26]. Despite the variations in age and methods utilized in assessing hip development, the importance of achieving proper hip centripetality remains unequivocal for optimal hip joint development. It is noteworthy that Chen et al. previously reported satisfactory outcomes with a CHDD of 6% or less one year post-reduction [5]. However, this finding is not entirely consistent with the results of our current study. Our study exclusively includes cases successfully treated with PH, whereas Chen et al.’s study encompassed cases that underwent both closed and open reductions. This difference in methodology, along with potential variations in patient demographics and outcome assessment techniques, may account for the discrepancies observed between the studies.

Moreover, AI is important for the good long-term result [8,19,27]. Albinana et al. reported that in hips with an AI greater than 30 degrees at four years post-reduction, 80% of the hips became Severin grade III/IV hip [8]. In this current report, there was no correlation between AI-4y and CHDDs. Similar to this study, Kitoh et al. also reported that there was no correlation between CHDD and acetabular development [28]. In contrast, Chen et al. and Kim et al. reported that CHDD most significantly correlated with acetabular development [5,25]. The observed discrepancies in results may be attributed to differences in patient demographics and reduction methods.

In this study, the lack of association between CHDD and AI might be due to the relatively short follow-up period. CHDD, being more dynamic, may improve more rapidly than AI. The modeling of the acetabulum might advance alongside improvements in CHDD. However, because the follow-up period in this study was short, it is possible that adequate acetabular modeling could not be observed. Furthermore, compared to AI, measurements of CHDD tend to exhibit higher intra- and inter-observer variability, which could potentially impact the observed relationship between CHDD and AI.

The limitations of this study include a reliance solely on plain radiographs for analysis, without incorporating advanced imaging techniques such as MRI or arthrography [26]. Furthermore, this investigation is characterized by a small sample size, a relatively short duration of follow-up, and a retrospective design. Furthermore, ΔCHDD (1-4y) represents the changes in CHDD from ages one to four, which means that it is not possible to calculate ΔCHDD (1-4y) until the child turns four years old. Therefore, predicting CHDD-4y at any point before that is difficult, and continuous monitoring is necessary until the child turns four years old.

## Conclusions

Some cases that showed improvement within the first year after reduction later deteriorated, while others that initially worsened within the first year subsequently improved. Changes in CHDD that occurred more than one year after reduction were found to influence the outcomes at a mean age of four years. Therefore, we suggest a follow-up period of at least four years is necessary for adequate assessment.
